# Neonatal Brain Metabolite Concentrations: An *In Vivo* Magnetic Resonance Spectroscopy Study with a Clinical MR System at 3 Tesla 

**DOI:** 10.1371/journal.pone.0082746

**Published:** 2013-11-28

**Authors:** Moyoko Tomiyasu, Noriko Aida, Mamiko Endo, Jun Shibasaki, Kumiko Nozawa, Eiji Shimizu, Hiroshi Tsuji, Takayuki Obata

**Affiliations:** 1 Research Center for Charged Particle Therapy, National Institute of Radiological Sciences, Chiba, Japan; 2 Department of Radiology, Kanagawa Children’s Medical Center, Yokohama, Japan; 3 Research Center for Child Mental Development, Chiba University Graduate School of Medicine, Chiba, Japan; 4 Department of Neonatology, Kanagawa Children’s Medical Center, Yokohama, Japan; Rikagaku Kenkyūsho Brain Science Institute, Japan

## Abstract

Brain metabolite concentrations change dynamically throughout development, especially during early childhood. The purpose of this study was to investigate the brain metabolite concentrations of neonates (postconceptional age (PCA): 30 to 43 weeks) using single-voxel magnetic resonance spectroscopy (MRS) and to discuss the relationships between the changes in the concentrations of such metabolites and brain development during the neonatal period. A total of 83 neonatal subjects were included using the following criteria: the neonates had to be free of radiological abnormalities, organic illness, and neurological symptoms; the MR spectra had to have signal-to-noise ratios ≥ 4; and the estimated metabolite concentrations had to display Cramér-Rao lower bounds of ≤ 30%. MRS data (echo time/repetition time, 30/5000 ms; 3T) were acquired from the basal ganglia (BG), centrum semiovale (CS), and the cerebellum. The concentrations of five metabolites were measured: creatine, choline, N-acetylaspartate, myo-inositol, and glutamate/glutamine complex (Glx). One hundred and eighty-four MR spectra were obtained (83 BG, 77 CS, and 24 cerebellum spectra). Creatine, N-acetylaspartate, and Glx displayed increases in their concentrations with PCA. Choline was not correlated with PCA in any region. As for myo-inositol, its concentration decreased with PCA in the BG, whereas it increased with PCA in the cerebellum. Quantitative brain metabolite concentrations and their changes during the neonatal period were assessed. Although the observed changes were partly similar to those detected in previous reports, our results are with more subjects (n = 83), and higher magnetic field (3T). The metabolite concentrations examined in this study and their changes are clinically useful indices of neonatal brain development.

## Introduction

*In vivo* magnetic resonance spectroscopy (MRS) of the brain can provide information about the concentrations of particular metabolites and changes in their levels, which can aid the early detection of abnormalities, e.g., in cases of acute neonatal brain injury in which diffusion imaging and conventional MR imaging (MRI) are negative [1]. The changes in the concentrations of brain metabolites throughout development have been extensively studied using MRS, and the concentrations of some metabolites have been found to exhibit dynamic changes during the early stages of life [1-13]. In children, it is necessary for detail classification of the metabolite concentrations depending on age. However, it is difficult to collect high quality MRS data from considerable number of healthy controls at each age group. The lack of the detail information about the normal concentration range depending on age sometimes makes difficult to perform disease assessment whether the changes are due to the disease or the development. 

At our children’s medical center, single-voxel MRS is usually included in routine clinical MR examinations of the brain and provides additional information for radiological diagnosis. Also in the neonates, hundreds of the MRS data are obtained per year. In this article, we focus on neonatal brain metabolite concentrations and their changes by the development. Neonatal subjects (postconceptional age (PCA): 30 to 43 weeks) with no radiological abnormalities, organic illnesses, or neurological symptoms were selected for this study. 　Note, the quality of the MRS data was also considered during the selection process. Then, the changes in their brain metabolite concentrations were investigated during the neonatal period. 

## Materials and Methods

### Ethics Statement

This retrospective study was approved by the institutional ethical review board of the Kanagawa Children’s Medical Center, where all of the clinical data in this study was acquired. Written informed consent was waived by the review board.

### Subjects

From October 2009 – November 2012, MRS examinations were performed on a total of 235 neonates, who ranged in PCA from 30 – 43 weeks, due to neurological symptoms such as hypoxic ischemic encephalopathy, a suspicion of organic disease, or as routine evaluations before discharge. The inclusion criteria were as follows: the subjects had to be free of radiological abnormalities, organic illness, and neurological symptoms at their latest radiological and clinical examinations. The MR images were interpreted by an experienced pediatric neuroradiologist and clinical and neurological conditions were evaluated by board-certificated neonatologists at our medical center. As a result, 79 neonates (males 36, females 43), including 60 preterm infants (gestational age (GA): 23 – 36 weeks; studied at PCA of 30 – 43 weeks; postnatal age (PNA): 39.9 ± 28.3 d) and 19 term infants (GA: 37 – 41 weeks; studied at PCA of 38 – 42 weeks; PNA: 7.1 ± 6.9 d) were included. Four of the 79 neonates were examined twice in different weeks, which resulted in a total of 83 sets of neonate MR spectra (males 38, females 45).

### Proton MRI and MRS

**Figure 1:**
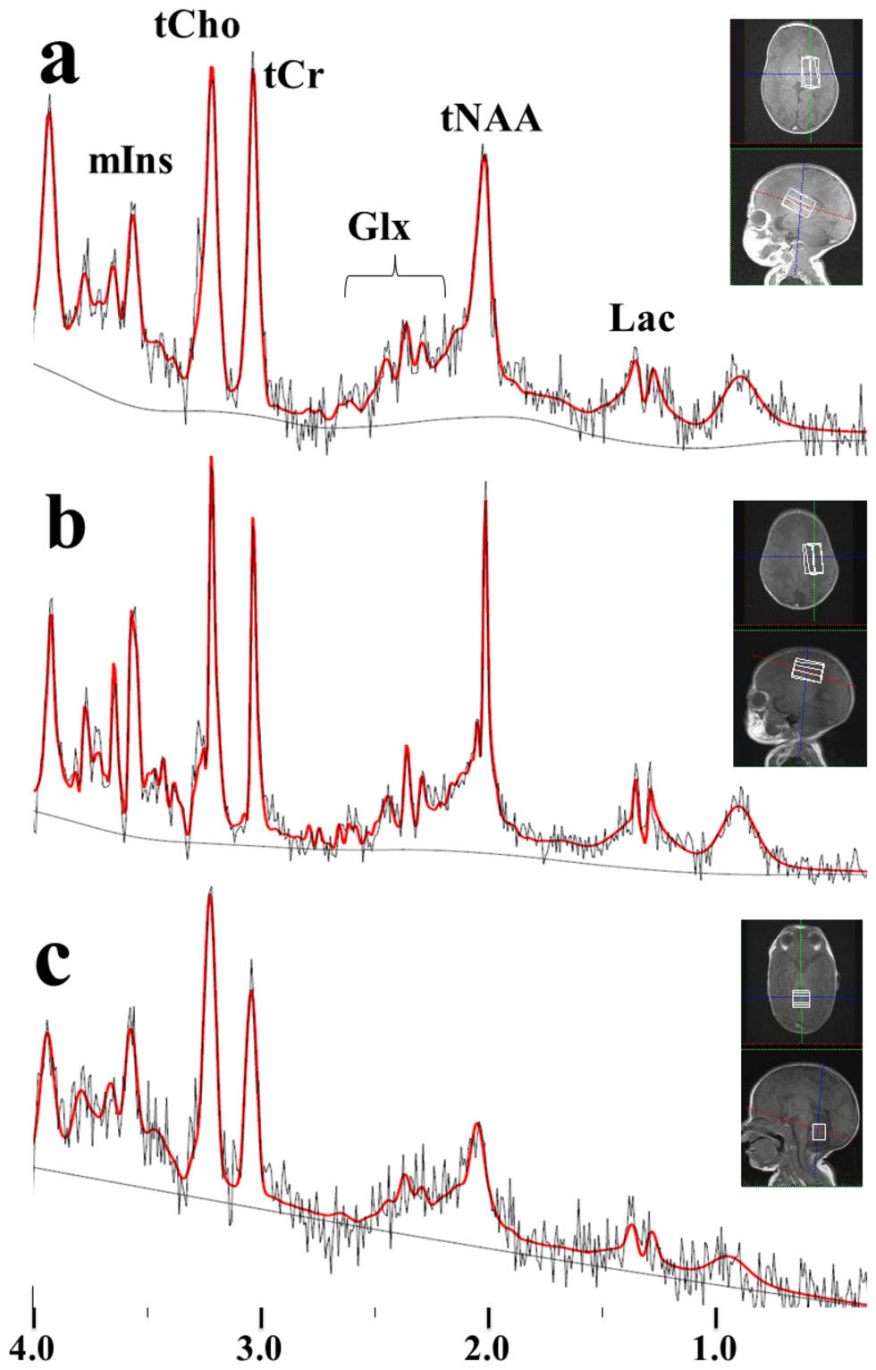
Representative LCModel outputs of *in* *vivo* proton MR spectra for the basal ganglia (a), centrum semiovale (b), and cerebellum (c) (volume, 4.2-6.5 mL; TE/TR, 30/5000 ms) obtained from a neonate at a postconceptional age of 37 weeks. Volumes of interest were imposed on the T1-weighted images. In the spectra, the bold lines represent the fitted lines produced by LCModel, and the thin lines are the original spectra.

All proton MRI and MRS scans were performed on a clinical 3T MR system (MAGNETOM Verio; Siemens, Erlangen, Germany) using a standard 12-element head coil (diameter: 25 cm) in the quadrature detection mode. During the examinations, the neonates were wrapped in vacuum-type immobilization bags (CFI Medical Solutions, Fenton, MI, USA) to prevent excessive head movement and protect their hearing. Sedation with thiopental (dose, 2 – 10 mg/kg of body weight) was employed where necessary (27 out of 83 neonates). During the examinations, heart rate and transcutaneous oxygen saturation were monitored continuously with a pulse oximeter (Nonin, Plymouth, MN, USA). For single-voxel MRS, the volume of interests (VOI) were placed in three regions: 1) the basal ganglia (BG) as a representative of the subcortical gray matter, 2) the centrum semiovale (CS) as a representative of the white matter, and 3) the cerebellum (Figure 1). T1-weighted (echo time (TE)/repetition time (TR): 9.4/400 ms) or T2-weighted (TE/TR: 120/5000 ms) MR images that had been obtained for clinical diagnosis were also used for the VOI positioning. The MRS was performed using a point-resolved spectroscopic localization sequence (PRESS) [14], a water presaturation pulse sequence, and the following parameters: TE/TR: 30/5000 ms; number of excitations: 4 to 32; spectral bandwidth: 1200 or 2000Hz; number of points: 1024. The excitation frequency was set to the proton resonance of water (4.7ppm, 123.24 MHz). The volume ranges of the BG, CS, and cerebellum were 2.4-6.7, 2.3-7.3, and 2.3-6.3 mL, respectively. Spectra of the same VOI were also obtained without the water presaturation pulse sequence using number of excitations of 2-4 and used to correct eddy-current-induced phase shifts and to quantify the subjects’ brain metabolite concentrations. It took about five minutes to obtain the pairs of MRS scans (with/without water presaturation) for each region; therefore, the total time required to perform MRS examinations of the three regions was about 15 min. 

The MRS data processing including the signal quantification was performed using LCModel (ver. 6.2-1G, Stephen Provencher Inc., Oakville, Ontario, Canada) [15,16] on a LINUX-based personal computer. To quantify the spectra, the “basis-set” of model spectra for the Siemens 3T PRESS sequence, which is internally defined in the LCModel package, was used as a reference to facilitate the separation of the spectra using linear combinations of individual metabolite spectra. The model spectra consisted of data for 16 reference compounds: alanine, aspartate, creatine (Cr), phosphorylcreatine (PCr), γ-aminobutyrate, glucose, glutamate (Glu), glutamine (Gln), glycerophosphorylcholine (GPC), phosphorylcholine (PCh), myo-inositol (mIns), lactate (Lac), N-acetylaspartate (NAA), N-acetyl-aspartyl-glutamate (NAAG), scyllo-inositol, and taurine. Metabolite concentrations were automatically calculated by comparing the proton peaks of the metabolites with that of unsuppressed water in the same VOI. The reference water concentration was assumed to be 46.9 M in all regions [5,10]. The signal-to-noise ratios (SNR) of the spectra and the percentage standard deviation (%S.D.) values of the metabolites were displayed in the LCModel output files. The SNR was defined as the ratio of the spectrum maximum minus the baseline divided by twice the root mean square residual, and the %S.D. was equal to the Cramér–Rao lower bound and was used as an index of the uncertainty in the metabolite concentration estimates [16]. The reliability of the data obtained in this study was ensured by excluding any data that was associated with an SNR of less than 4 or a %S.D. value of more than 30. These are similar protocol to our previous studies [17-19].

In this study, the concentrations of five metabolites were assessed: tCr, the total concentration of Cr and PCr; tCho, the total concentration of GPC (including other choline-containing compounds [16]) and PCh; tNAA, the total concentration of NAA and NAAG; mIns; and Glx, the total concentration of Glu and Gln complex. In the statistical analysis, Pearson’s correlation coefficients were calculated for the relationships between the concentration of each metabolite and PCA. Then, regression line equations were calculated for the statistically significant relationships. The independent samples *t*-test was used for group comparisons of metabolite concentrations between preterm infants at term (PT) and full term infants at term (FT). For the comparisons of the metabolites between the sexes, the analysis of covariance (ANCOVA) with covariate of PCA was used. *P*-values of less than 0.05 were considered to be significant. 

## Results

In the neonatal MR spectra, the five target metabolites (tCr, tCho, tNAA, mIns, and Glx) were clearly observed, and Lac doublet peaks were also frequently detected (Figure 1). There were no significant differences in the concentrations between the sexes (ANCOVA). 

In total, 184 MR spectra (83 BG, 77 CS, and 24 cerebellum spectra) were obtained from the 83 neonates. The numbers of each metabolite to meet the quality criteria were as follows: tCr (83 BG, 77 CS, and 24 cerebellum); tCho (83, 77, and 24); tNAA (83, 76, and 14); mIns (83, 77, and 23); Glx (79, 73, and 17).

**Figure 2:**
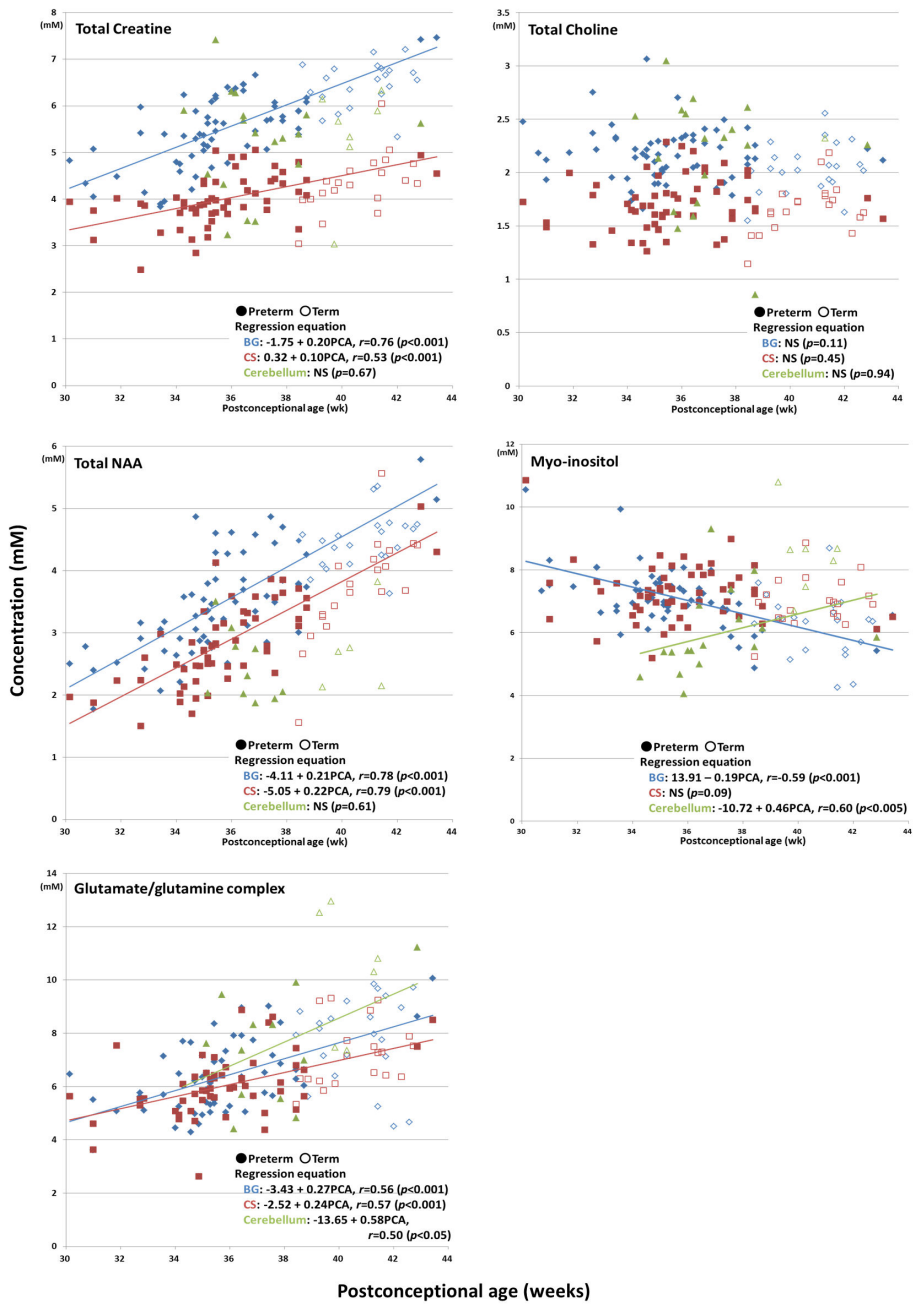
Scatterplots of the changes in metabolite concentrations with neonatal postconceptional age (weeks); the basal ganglia: diamonds, centrum semiovale: rectangles, and cerebellum: triangles.

tCr, tNAA, and Glx displayed increases in their concentrations with PCA (Figure 2). Among them, the concentration of tNAA demonstrated the greatest increases; i.e., it increased by over two-fold during the period from 30 to 43 weeks PCA in the BG and CS. tCho did not exhibit any correlations with PCA in any region. As for mIns, its concentration decreased with PCA in the BG, whereas it increased with PCA in the cerebellum. Regarding all of the target metabolites except mIns, their concentration levels in the BG tended to be higher than those in the CS throughout the neonatal period (Figure 2).

There were no significant differences in the levels of any metabolite between the PT and FT (t-test, Table 1). However, no comparisons between these two groups were performed for the cerebellum due to the small sample sizes for this region (PT, n = 4; FT, n = 7). 

**Table 1:** Brain metabolite concentrations of individuals with almost identical postconceptional age (PCA) values but different postnatal age (PNA) values.

		PCA (wk)	PNA (d)	tCr (mM)	tCho (mM)	tNAA (mM)	mIns (mM)	Glx (mM)
Basal ganglia	Preterm infants at term (n =7)	39.9 (2.3)	85.7 (16.7)	6.3 (0.8)	2.2 (0.1)	4.3 (0.9)	6.1 (0.8)	7.0 (1.6)
	Full term infants (n = 22)	40.7 (1.3)	7.1 (6.9)	6.4 (0.5)	2.0 (0.2)	4.4 (0.4	6.3 (1.0)	7.8 (1.6)
	*p* value		< 0.001	0.51	0.11	0.74	0.65	0.62
Centrum Semiovale	Preterm infants at term (n =7)	39.9 (2.3)	85.7 (16.7)	4.3 (0.5)	1.8 (0.2)	3.8 (0.7)	6.9 (0.7)	6.8 (1.2)
	Full term infants (n = 20)	40.6 (1.3)	6.9 (7.2)	4.3 (0.6)	1.7 (0.2)	3.7 (0.8)	7.0 (0.8)	7.2 (1.2)
	*p* value		< 0.001	0.94	0.33	0.92	0.86	0.43

Data denote the averages of the values, and ± standard deviations are in parentheses.

## Discussion

In this study, we quantitatively assessed neonatal brain metabolite concentrations (tCr, tCho, tNAA, mIns, and Glx) in the BG, CS, and cerebellum using short TE (30 ms) MRS, which was performed with a clinical 3T scanner. To the best of our knowledge, this is the first such study to include a reasonably large number of neonates (n = 83) without radiological or short-term clinical abnormality, with reasonably high MRS data quality (SNR ≥ 4, and %S.D. ≤ 30). Furthermore, we acquired MR spectra not only from the cerebrum but also from the small neonatal cerebellum, and our all MRS data were obtained on 3T MR system with higher SNR and greater spatial resolution than 1.5T; therefore, our data would provide novel and/or more precious information about the metabolite concentrations and their developmental changes in the neonatal brain.

### Metabolite concentration changes with PCA

tNAA (NAA + NAAG) showed marked increases in its concentration during the neonatal period, as was reported previously [1,2,4-7,9-13]. NAA is produced in neuronal mitochondria and plays a role in myelination as a source of acetyl groups for lipid synthesis. NAA also serves as a precursor for the synthesis of NAAG, which acts as a cotransmitter in combination with several other neurotransmitters [1,9,12,20,21]. It was found that the concentration of NAA increased markedly during the period from 32 - 42 weeks gestation; i.e., prior to the initiation of myelination. Such dynamic NAA increases during this period might reflect the use of acetyl groups for myelin deposition [3-7,9].

tCr acts as a “battery” for replenishing ATP levels [1,5,12,22]. In adults, its concentration usually remains relatively stable during aging as well as in a variety of diseases, and hence, it is sometimes used as an internal reference for other metabolites [22]. However, as has been reported in previous neonatal studies, the concentration of tCr increased markedly with PCA in the present study, which might have been due to the increasing energy demand associated with cerebral maturation [5,6,10,12].

tCho is involved in membrane synthesis in the brain and acts as a precursor for the neurotransmitter acetylcholine, which has many functions in the nervous system [1,12,22,23]. Our study did not detect any correlations between the concentration of tCho and PCA in the BG, CS, or cerebellum, and similar results have been reported previously; i.e., no correlations between these parameters were detected in the gray matter, thalamus, or white matter during the period from 32 - 43 weeks PCA [10] or in the motor cortex region during the period from 33 - 42 weeks PCA [6], and no concentration differences were observed between preterm and term infants in the precentral area [7] or cerebellum [4]. On the other hand, tCho displays higher concentrations during early life than in adulthood, indicating that choline-containing compounds are turned over more rapidly during early human development [1,4-6,10]. Brighina et al. reported that the most prominent reduction in the concentration of tCho occurs after the age of 2, by which point myelination has almost been completed, indicating that tCho is progressively incorporated into the lipids that make up myelin [5,12]. 

The peak observed at 3.56 ppm, which was considered to represent mIns, is also known to contain glycine [22], and it is sometimes difficult to determine the individual concentrations of these metabolites. However, it is likely that mIns is the main component of this peak [5,10] and so the glycine component was not considered in this study. mIns has many functions, for example, it plays roles in osmotic regulation and cellular nutrition and forms complexes such as inositol-1-phosphateand phosphatidyl inositol [1,5,11,12]. Therefore, it is sometimes difficult to identify which concentration increases/decreases are caused by particular functional changes. In this study, decreases in the concentration of mIns were observed in the BG. Marked reductions in the concentration of mIns have also been reported in previous neonatal studies; however, the cause of these changes remains unclear [1,5,6,9-12]. Kreis et al. [5] suggested that mIns plays a role in surfactant production in neonates. In addition, they found that in some supratentorial regions its concentration decreased rapidly after birth, and these reductions appeared to be associated with PNA rather than GA. However, in our analyses we found that the mIns concentration was more strongly correlated with GA than PNA (data not shown). On the other hand, Roelants-van Rijn et al. [11] did not detect any association between the concentration of mIns and PNA, suggesting that reductions in the brain concentrations of mIns might be due to the decreases in brain water levels that accompany changes in the osmoregulation of the brain. Further studies are needed to clarify the changes in the concentration of mIns that occur during the neonatal period.

A novel finding of the present study was the increase in the concentration of mIns observed in the cerebellum (Figure 2, *p* < 0.005) with more sample size (n = 23) and higher magnetic field (3T) than previous studies: Hüppi et al. [4] did not observe any difference in the concentration of mIns between preterm and term infants. It was reported that cerebellar volume increased by about four-fold from birth to 12 months, whereas that of the whole brain was about two-fold [24,25]. Thus, the increase in the mIns concentration observed in the cerebellum might be more closely associated with the late-onset of brain development than the changes seen in the supratentorial region. 

Glu is an excitatory neurotransmitter, and Gln is involved in Glu detoxification, osmolyte synthesis, and ammonia detoxification [1,12,22,26]. Glx concentration increases have been detected during the neonatal period in previous studies [10,11]. As was suggested for tNAA and tCr, the large increase in the Glx concentration observed during the progression of PCA might have been associated with cerebral maturation. 

Regarding the cerebellum, only 24 out of the 83 MR spectra were included in the analysis because the rest had low SNR and/or high %S.D. values, mainly due to the small volume of the cerebellum (about 26.9 cm^3^ even at full term) [27]. However, since our data plots (Figure 2) showed significant dynamic changes in the concentration of mIns with PCA, despite the small sample size, mIns might be useful for evaluating neonatal brain development.

None of the metabolites displayed concentration differences between the PT and FT (Table 1), which agrees with the findings of Hüppi et al. [6,7]. Therefore, for preterm infants metabolite concentrations might be useful indices of brain development, although other factors such as brain volume also need to be evaluated [28,29].

In this study, the Lac concentration was not analyzed quantitatively (for the reason, see *Limitations*); however, Lac peaks were frequently observed (Figure 1), as has been reported previously [9,10,12,13,30-32]. It was suggested that Lac might be produced in astrocytes and then taken up by neurons and metabolized into energy, which is known as the Lac shuttle [9,12,26,33,34]. Pellerin et al. [33] reported that the Lac shuttle is much more active in the immature brain, which could explain the higher lactate peaks detected by proton MRS in the immature normal brain.

### Limitations

#### Subjects

It is possible that some of the neonatal subjects were not completely healthy. However, the subjects were carefully selected and have since been followed-up by medical doctors at our center. At present (June 2013), the subjects have reached corrected ages of 6 months to 3 years. If a subject displayed any abnormalities, such as genetic abnormalities or a reduced developmental quotient, during the follow-up examinations, then their MRS data were excluded from the study.

#### Water concentration and relaxation times

The relaxation times and concentration of water change during development, even during the neonatal period [35]. However, it was not practical for us to obtain accurate values for these parameters by performing more MRI examinations. Instead, a constant water concentration value of 46.9 M was used in this study [5,10]. No corrections for the T1/T2 relaxation times were applied to the LCModel outputs. Compared with adults, the water relaxation times of the neonates were longer in both the T1 (2254 ms in the BG, 2934 ms in the CS) and T2 (137 ms in the BG, 265 ms in the CS; 80 ms in the LCModel setting) [16,35]. We could not find any T1/T2 reference values for 3T MRS of the cerebellum in previous studies. Using a TE/TR of 30/5000 ms, the metabolite concentrations were over- and underestimated without considering the T1 and T2, respectively. However, the errors counteracted each other, and the metabolite concentrations were estimated to be 4% and 6% higher than the uncorrected values in the BG and CS, respectively. Also, the T2 errors in each metabolite were assumed to have a maximum effect of about 2% on the metabolite concentration values [5]. Moreover, in this period, any changes in the T1 or T2 relaxation times of water protons, or the T2 metabolite values would have been very small; therefore, the uncorrected data were considered to be sufficiently reliable for analyzing the correlations between metabolite levels and PCA. 

#### Low concentration metabolites

Due to our selection criteria, only five metabolites were evaluable. Data for metabolites with low concentrations often display large %S.D. values; i.e., a large degree of uncertainty regarding the existence of the peak. When a metabolite displays a concentration of 0, its %S.D. value is infinite. We often observed Lac peaks; however, quite a few Lac peaks showed large %S.D. values; i.e., there was a greater possibility that the peaks were actually noise rather than Lac peaks, and so had to be excluded from the analysis. Therefore, it is often difficult to obtain publishable results for such metabolites, even if the changes in their concentrations seem to be important. Some advanced methods such as spectral editing and two-dimensional proton techniques may provide accurate information about the low SNR metabolites [36,37].

## Conclusions

Quantitative brain metabolite concentrations and their changes during the neonatal period were assessed. Although our findings were partly similar to those of previous studies, such as concentration increasing trends with development in some metabolites, our results are more quantitative with more subjects (n = 83) and higher magnetic field (3T). Data regarding the ‘absolute’ concentrations of brain metabolites are also valuable because the concentration of each metabolite changes in a different manner, and it would be difficult to determine the changes in the levels of a particular metabolite from the changes in its concentration relative to those of other metabolites. The metabolite concentrations examined in this study and their changes are clinically useful indices of neonatal brain development. Further studies will increase our understanding about such brain metabolites including their specific functions during the neonatal period.
